# MicroRNAs in obesity, sarcopenia, and commonalities for sarcopenic obesity: a systematic review

**DOI:** 10.1002/jcsm.12878

**Published:** 2022-01-04

**Authors:** Lisa Dowling, Ankita Duseja, Tatiane Vilaca, Jennifer S. Walsh, Katarzyna Goljanek‐Whysall

**Affiliations:** ^1^ The University of Sheffield Sheffield UK; ^2^ The University of Liverpool Liverpool UK; ^3^ Department of Physiology, School of Medicine, Nursing and Health Sciences, College of Medicine National University of Ireland, Galway Galway Ireland

**Keywords:** MicroRNA, Sarcopenia, Obesity, Frailty, Metabolic syndrome

## Abstract

Sarcopenic obesity is a distinct condition of sarcopenia in the context of obesity, with the cumulative health risks of both phenotypes. Differential expression of microRNAs (miRNAs) has been reported separately in people with obesity and sarcopenia and may play a role in the pathogenesis of sarcopenic obesity. However, this has not been explored to date. This study aimed to identify differentially expressed miRNAs reported in serum, plasma, and skeletal muscle of people with obesity and sarcopenia and whether there are any commonalities between these conditions. We performed a systematic review on Embase and MEDLINE (PROSPERO, CRD42020224486) for differentially expressed miRNAs (fold change >1.5 or *P*‐value <0.05) in (i) sarcopenia or frailty and (ii) obesity or metabolic syndrome. The functions and targets of miRNAs commonly changed in both conditions, in the same direction, were searched using PubMed. Following deduplication, 247 obesity and 42 sarcopenia studies were identified for full‐text screening. Screening identified 36 obesity and 6 sarcopenia studies for final inclusion. A total of 351 miRNAs were identified in obesity and 157 in sarcopenia. Fifty‐five miRNAs were identified in both obesity and sarcopenia—by sample type, 48 were found in plasma and one each in serum and skeletal muscle. Twenty‐four miRNAs were identified from 10 of the included studies as commonly changed in the same direction (22 in plasma and one each in serum and skeletal muscle) in obesity and sarcopenia. The majority of miRNA‐validated targets identified in the literature search were members of the phosphoinositide 3‐kinase/protein kinase B and transforming growth factor‐β signalling pathways. The most common targets identified were insulin‐like growth factor 1 (miR‐424‐5p, miR‐483‐3p, and miR‐18b‐5p) and members of the SMAD family (miR‐483‐3p, miR‐92a‐3p, and miR‐424‐5p). The majority of commonly changed miRNAs were involved in protein homeostasis, mitochondrial dynamics, determination of muscle fibre type, insulin resistance, and adipogenesis. Twenty‐four miRNAs were identified as commonly dysregulated in obesity and sarcopenia with functions and targets implicated in the pathogenesis of sarcopenic obesity. Given the adverse health outcomes associated with sarcopenic obesity, understanding the pathogenesis underlying this phenotype has the potential to lead to effective screening, monitoring, or treatment strategies. Further research is now required to confirm whether these miRNAs are differentially expressed in older adults with sarcopenic obesity.

## Introduction

Sarcopenic obesity is a condition of excess fat mass and sarcopenia.^1^, ^2^ Differing definitions of sarcopenia have been proposed with growing consensus on the importance of muscle function.^1^, ^3^ Sarcopenic obesity is more commonly found amongst older adults; however, it can also be found in younger adults during both acute and chronic disease, or intermittent weight cycling.^4^ Dependent on the definition used, sarcopenic obesity is thought to range in prevalence from 2.75% to over 20%.^4^ Of clinical importance, sarcopenic obesity may have the cumulative risk of both sarcopenia and obesity.^5^ Growing evidence supports this with a greater risk of falls, hospitalization, worsening disability, and all‐cause mortality reported.^6^, ^7^, ^8^

The aetiology of sarcopenic obesity is complex and not fully understood (see Batsis and Villareal and Zamboni *et al*.^2^, ^9^ for detailed reviews). Ageing is associated with changes in body composition including a loss of lean mass, increased body fat, and muscular fat infiltration, with a subsequent reduction in resting metabolic rate.^2^, ^10^ Reduced physical activity and malnutrition (including overnutrition or undernutrition and malabsorption) associated with ageing contribute to a gradual increase in body fat^2^ and the development of sarcopenia.^1^ Moreover, excess body fat or obesity can exacerbate sarcopenia.^1^, ^11^ Obesity is associated with low‐grade inflammation with the secretion of tumour necrosis factor, leptin, and C‐reactive protein.^2^, ^12^ Leptin elevates the levels of pro‐inflammatory cytokines, which cause a reduction in the anabolic effects of insulin‐like growth factor 1 (IGF‐1).^2^ This inflammation leads to insulin resistance, further exacerbated by muscle catabolism, which promotes fat mass and loss of muscle mass.^2^, ^12^ As such, changes associated with ageing, obesity, and sarcopenia as well as interrelationships between these phenotypes can contribute to the pathogenesis of sarcopenic obesity.

MicroRNAs (miRNAs, miRs) are short, non‐coding RNAs that can regulate gene expression at a post‐transcriptional level.^13^ To date, 2654 miRNAs have been discovered, which are predicted to regulate two‐thirds of the human genome.^14^, ^15^ Therefore, many miRNAs may modulate many physiological processes. There is evidence that ageing changes miRNA levels in the muscle and that these changes may have a detrimental impact on muscle quality and quantity.^16^, ^17^ However, the influence of obesity or adiposity on the miRNA profile of older adults and whether this translates into functional impairment has not yet been established. Evidence from rodent studies has demonstrated that adipose‐derived miRNAs can be transported via exosomes to a variety of host cells including myocytes, hepatocytes, and macrophages.^18^, ^19^, ^20^ Likewise, skeletal muscle‐derived miRNAs can be taken up by adipose tissue.^21^ Functionally, this inter‐organ crosstalk has been implicated in insulin resistance, adipogenesis, and lipid metabolism,^18^, ^21^ thus suggesting a role for miRNAs in the pathogenesis of sarcopenic obesity. MiRNAs are an exciting area of research due to the potential of antagomiRs, which are already been explored as pharmacological options in conditions such as cardiovascular disease and cancer.^22^, ^23^

The primary aim of this systematic review was to identify differentially expressed miRNAs reported in plasma, serum, or skeletal muscle of adults with obesity or sarcopenia to determine common miRNA changes between these phenotypes. As this is an emerging area with limited research, studies reporting (i) sarcopenia or frailty and (ii) obesity or metabolic syndrome were included because of similarities between definitions. A secondary aim of this review was to identify the targets and functions of these differentially expressed miRNAs.

## Methods

### Protocol registration

The Preferred Reporting Items for Systematic Reviews and Meta‐Analyses (PRISMA) statement was followed as a reference protocol standard.^24^ A PRISMA flow chart is included. Our protocol was registered at the International Prospective Register of Systematic Reviews PROSPERO, with Registration Number CRD42020224486; available at https://www.crd.york.ac.uk/prospero/display_record.php?ID=CRD42020224486.

### Bibliographical search and eligibility criteria

This systematic review consisted of two searches performed on MEDLINE and Embase (last searched 6 January 2021). Part 1 searched for studies of sarcopenia or frailty using the following terms: MicroRNA/miR/miRNA AND ‘sarcopenia’, ‘muscle strength’, ‘frail’, ‘ageing’/‘older adult’/‘aged’ AND ‘muscle’, ‘muscle weakness’, OR ‘dynapenia’. Part 2 searched for studies of obesity or metabolic syndrome using the following terms: MicroRNA/miR/miRNA combined with ‘obesity’ OR ‘metabolic syndrome’. The multipurpose function was used for keywords, and MeSH terms were used where available. Articles were limited to the English language and human studies. Eligible studies enrolled adult participants (>18 years) with sarcopenia, frailty, metabolic syndrome, or obesity and comparable non‐sarcopenic/frail or non‐obese/metabolic syndrome controls as outlined in Supporting Information, *Table* S1. Studies were excluded if the primary condition of interest was not sarcopenia, frailty, metabolic syndrome, or obesity but instead an unrelated disease or condition, for example, type 1 diabetes, cancer, or pregnancy, which may have confounded findings. Study groups containing some, but not all, type 2 diabetes participants were included. The considered biological fluids and tissues were serum, plasma, and skeletal muscle. Results on tissue samples or cell lines were excluded. Observational (cohort and case control) studies or intervention studies with relevant baseline results were included.

### Study selection

Following deduplication, all selected titles and abstracts were screened to identify articles for full‐text screening. A second reviewer verified a random sample of articles included in the first sift. The second sift, which consisted of full‐text screening, was independently conducted by two reviewers to confirm that the criteria for the condition of interest, samples, age group, and outcome measure in the study met eligibility criteria. In cases where the same or similar results were reported in more than one study, the publication with the most information was included and the other rejected for duplication. Authors of papers with insufficient information relating to eligibility criteria or outcome measures for this review were contacted. If a reply was not received within one month, only the information reported in the paper was included (e.g. incomplete list of miRNAs) or else the paper was rejected if eligibility criteria remained unclear (e.g. age group). A more comprehensive list of miRNA results was obtained from one study following email correspondence.^25^ Disagreements between reviewers that could not be solved with discussion were resolved with a third reviewer by consensus.

### Data extraction

A standardized form was used to extract trial features (authors, published year, and country), patient characteristics (age and sex), RNA extraction and detection methods, and the subset of differentially expressed miRNAs between the two conditions. Extracted information was verified by a second reviewer.

### Risk of bias in individual studies

Two reviewers assessed the quality and risk of bias of individual studies using the Newcastle–Ottawa Scale (NOS) with an additional star for validation of results within the study using either two measurement methods or two study groups (*Table* S2). Because of the type of studies included, all studies were given a star for the question on non‐response rate. Disagreements between reviewers that could not be solved through discussion were resolved with a third reviewer by consensus.

### Summary measures

The outcome measure was differentially expressed miRNAs in human skeletal muscle, plasma, or serum with at least a 1.5‐fold change or *P* < 0.05 measured using RT‐qPCR, next‐generation sequencing, or microarray. Circulating (serum and plasma) miRNAs may be useful as non‐invasive biomarkers, whereas miRNAs found in muscle may provide a mechanistic insight into sarcopenic obesity.

Because of differences in nomenclature between studies, we used the information for previous miRNA IDs on miRBase (Release 22.1; October 2018) to clarify and update the nomenclature of included miRNAs, which did not specify whether they were ‐3p or ‐5p.^15^ The BioVenn online interface was then used to identify potentially overlapping miRNAs.^26^ Overlapping miRNAs that were differentially expressed in both obesity and sarcopenia were further classified by tissue/fluid type and as either differentially expressed in (i) the same direction (e.g. up‐regulated), (ii) different directions (e.g. up‐regulated in obesity and down‐regulated in sarcopenia), (iii) conflicting directions (e.g. both up‐regulated and down‐regulated in one condition but not the other), or (iv) unclear (e.g. differences in nomenclature limited interpretation).

### Synthesis of results and additional analyses

MicroRNAs that were differentially expressed in the same direction in obesity and sarcopenia were identified for further investigation. A literature search was conducted on PubMed to identify validated target genes or functions of these miRNAs with regard to muscle, sarcopenia or frailty and obesity, metabolic syndrome, or insulin resistance. A narrative synthesis of the findings from the studies and in the context of sarcopenic obesity, their target genes, and metabolic pathways implicated is provided.

## Results

### Bibliographical search

**Figure 1:**
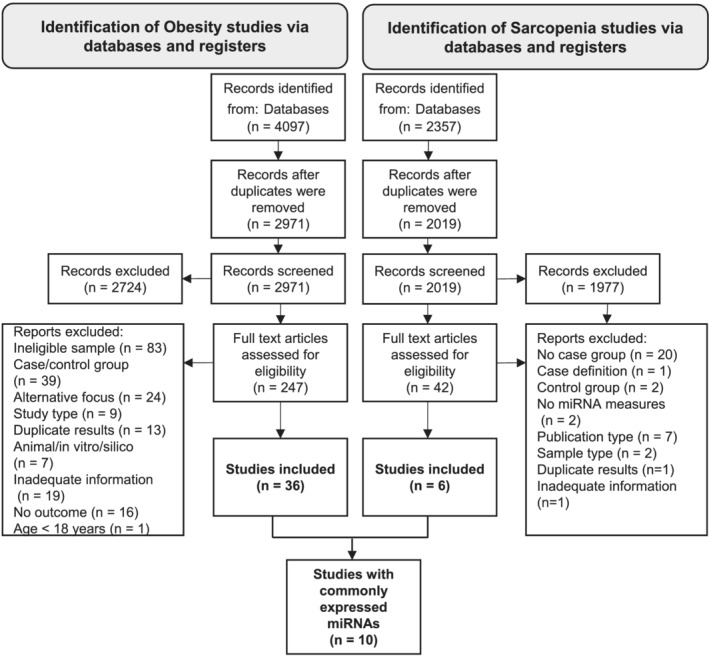
PRISMA flow chart for obesity/metabolic syndrome and sarcopenia/frailty parts of the systematic review.

The bibliographical search in MEDLINE and Embase retrieved 4097 obesity‐related papers and 2357 sarcopenia‐related papers published before 6 January 2021. Following deduplication, 2971 papers were screened for obesity and 2019 for sarcopenia. Following screening, 247 obesity studies and 42 sarcopenia studies were included for full‐text review. In total, 36 studies were identified for obesity and six for sarcopenia (*Figure* 1). MiRNAs dysregulated in both sarcopenia and obesity were identified from 10 studies.^25^, ^27^, ^28^, ^29^, ^30^, ^31^, ^32^, ^33^, ^34^, ^35^

Studies were conducted in Korea,^25^ China,^32^ Singapore,^29^ New Zealand,^28^, ^30^ the USA,^31^, ^34^ Spain,^27^ and the UK.^33^ The majority of obesity studies used World Health Organization criteria for obesity,^25^, ^27^, ^29^, ^30^ although the criteria for two studies were unclear.^28^, ^35^ The sarcopenia studies used Fried Frailty Score,^31^ Asian Working Group for Sarcopenia,^32^ and European Working Group on Sarcopenia in Older People 2010^33^, ^34^ criteria. Four studies were conducted in women only^27^, ^28^, ^30^, ^34^ and three studies in men only.^29^, ^33^, ^35^ Two studies recruited both men and women,^25^, ^31^ and one study did not report the sex of participants.^32^ Based on the reported average age, obese participants would not be defined as older adults, age ≥65 years.^25^, ^27^, ^28^, ^29^, ^30^ Sarcopenia studies recruited older adults,^31^, ^32^, ^33^ although one used a younger cut‐off of 60–85 years.^34^ Only three studies validated their findings.^27^, ^30^, ^32^

### MicroRNAs reported as dysregulated in the context of sarcopenia and obesity

**Figure 2:**
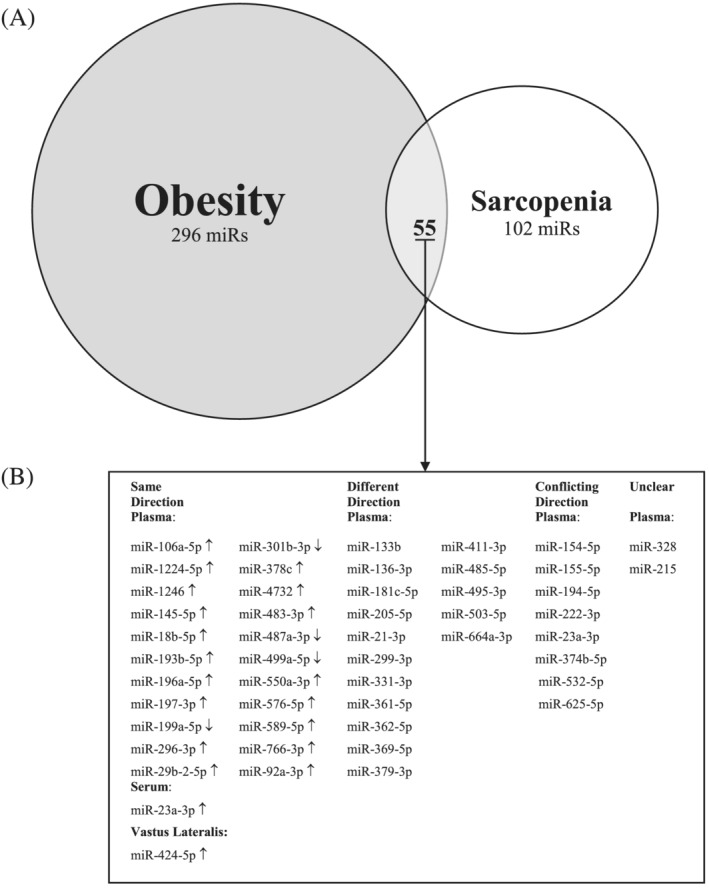
(A) Venn diagram of miRNAs commonly expressed in all tissues in both obesity and sarcopenia. (B) MiRNAs by sample type (plasma, serum, or vastus lateralis) found in both obesity and sarcopenia. ‘↑’ refers to overexpressed; ‘↓’ refers to underexpressed. Since the publication of several studies included in this review, some reported miRs have been removed from the latest version of miRBase (e.g. miR‐4461, miR‐4532, and miR‐6087); this does not affect overlapping miRs.

A total of 351 miRNAs were identified in obesity and 157 in sarcopenia (*Figure* 2). Fifty‐five potential miRNAs were identified in both obesity and sarcopenia. When examined by sample type, 48 overlapping miRNAs were identified in plasma and one each in serum and skeletal muscle (vastus lateralis). Sixteen plasma miRNAs were expressed in differing directions in obesity and sarcopenia. Eight plasma miRNAs in obesity, which were also present in sarcopenia, were expressed in conflicting directions. Two plasma miRNAs could not be determined with confidence because of the nomenclature in the studies (miR‐328 and miR‐215). Therefore, across six obesity^25^, ^27^, ^28^, ^29^, ^30^, ^35^ and four sarcopenia^31^, ^32^, ^33^, ^34^ studies, we manually identified 24 miRNAs differentially expressed in the same direction. Twenty‐two of these miRNAs were found in plasma and one each in serum and skeletal muscle (*Table* 1). Of these overlapping miRNAs, only miR‐23a‐3p was reported in more than one tissue (serum and plasma) of both sarcopenia and obesity; however, in plasma, conflicting directions were reported in obesity. The majority of overlapping miRNAs were identified in two studies, one of which used RT‐qPCR^29^ and the other used RNA‐Seq.^31^ Exosomal miRNAs were reported by three studies.^25^, ^27^, ^31^ Twenty‐one of the 24 overlapping miRNAs were found in one study of frailty using plasma exosomes.^31^

**Table 1:** Summary characteristics of studies with overlapping miRNAs in the same direction

		Obese					Sarcopenia				
	MiRNA	Country	Obesity definition	*N* (%female)	Age (years)	Log2FC	Country	Sarcopenia definition	*N* (%female)	Age (years)	Log2FC
	**Plasma**										
1	MiR‐106a‐5p	Spain^27^	BMI ≥ 30 kg/m^2^	Ob 12 (100%) Lean 19 (100%)	Range 30–70 49.9 ± 11.3 45.1 ± 15.1	ND (Up)	USA^31^	Fried Frailty Phenotype	Fr 7 (0%) N‐F 7 (71%)	Range 71–89 85.6 ± 3.8 76 ± 6.5	1.81
2	MiR‐18b‐5p	New Zealand^28^	ND	Ob 11 (100%) Lean 12 (100%)	41 ± 5 44 ± 9	ND (Up)	USA^31^	Fried Frailty Phenotype	Fr 7 (0%) N‐F 7 (71%)	Range 71–89 85.6 ± 3.8 76 ± 6.5	4.00
3	MiR‐193b‐5p	Singapore^29^	BMI ≥ 27.5 kg/m^2^	Ob 9 (0%) Lean 9 (0%)	28.4 ± 1.6^a^ 23.2 ± 0.2^a^	1.89	USA^31^	Fried Frailty Phenotype	Fr 7 (0%) N‐F 7 (71%)	Range 71–89 85.6 ± 3.8 76 ± 6.5	1.96
4	MiR‐197‐3p	New Zealand^28^	ND	Ob 11 (100%) Lean 12 (100%)	41 ± 5 44 ± 9	ND (Up)	USA^31^	Fried Frailty Phenotype	Fr 7 (0%) N‐F 7 (71%)	Range 71–89 85.6 ± 3.8 76 ± 6.5	1.85
5	MiR‐199a‐5p	Singapore^29^	BMI ≥ 27.5 kg/m^2^	Ob 9 (0%) Lean 9 (0%)	28.4 ± 1.6^a^ 23.2 ± 0.2^a^	−1.324	USA^31^	Fried Frailty Phenotype	Fr 7 (0%) N‐F 7 (71%)	Range 71–89 85.6 ± 3.8 76 ± 6.5	−0.74
6	MiR‐483‐3p	Singapore^29^	BMI ≥ 27.5 kg/m^2^	Ob 9 (0%) Lean 9 (0%)	28.4 ± 1.6^a^ 23.2 ± 0.2^a^	1.413	USA^31^	Fried Frailty Phenotype	Fr 7 (0%) N‐F 7 (71%)	Range 71–89 85.6 ± 3.8 76 ± 6.5	3.92
7	MiR‐499	New Zealand^28^	BMI > 30 kg/m^2^	Ob 80 (100%) Lean 80 (100%)	52.5 ± 10.5^b^ 53.0 ± 13.5^b^	ND (Down)	China^32^	AWGS	S 93 (ND) N‐S 93 (ND)	≥65 76.15 ± 0.58^a^ 76.19 ± 0.58^a^	Down
8	MiR‐550a‐3p	Singapore^29^	BMI ≥ 27.5 kg/m^2^	Ob 9 (0%) Lean 9 (0%)	28.4 ± 1.6^a^ 23.2 ± 0.2^a^	0.782	USA^31^	Fried Frailty Phenotype	Fr 7 (0%) N‐F 7 (71%)	Range 71–89 85.6 ± 3.8 76 ± 6.5	3.08
9	MiR‐576‐5p	Singapore^29^	BMI ≥ 27.5 kg/m^2^	Ob 9 (0%) Lean 9 (0%)	28.4 ± 1.6^a^ 23.2 ± 0.2^a^	0.501	USA^31^	Fried Frailty Phenotype	Fr 7 (0%) N‐F 7 (71%)	Range 71–89 85.6 ± 3.8 76 ± 6.5	3.24
10	MiR‐589‐5p	Singapore^29^	BMI ≥ 27.5 kg/m^2^	Ob 9 (0%) Lean 9 (0%)	28.4 ± 1.6^a^ 23.2 ± 0.2^a^	0.274	USA^31^	Fried Frailty Phenotype	Fr 7 (0%) N‐F 7 (71%)	Range 71–89 85.6 ± 3.8 76 ± 6.5	1.81
11	MiR‐92a‐3p	Singapore^29^	BMI ≥ 27.5 kg/m^2^	Ob 9 (0%) Lean 9 (0%)	28.4 ± 1.6^a^ 23.2 ± 0.2^a^	0.571	USA^31^	Fried Frailty Phenotype	Fr 7 (0%) N‐F 7 (71%)	Range 71–89 85.6 ± 3.8 76 ± 6.5	0.78
12	MiR‐1224‐5p	Singapore^29^	BMI ≥ 27.5 kg/m^2^	Ob 9 (0%) Lean 9 (0%)	28.4 ± 1.6^a^ 23.2 ± 0.2^a^	0.987	USA^31^	Fried Frailty Phenotype	Fr 7 (0%) N‐F 7 (71%)	Range 71–89 85.6 ± 3.8 76 ± 6.5	0.49
13	MiR‐1246	Singapore^29^	BMI ≥ 27.5 kg/m^2^	Ob 9 (0%) Lean 9 (0%)	28.4 ± 1.6^a^ 23.2 ± 0.2^a^	1.254	USA^31^	Fried Frailty Phenotype	Fr 7 (0%) N‐F 7 (71%)	Range 71–89 85.6 ± 3.8 76 ± 6.5	3.23
14	MiR‐145‐5p	New Zealand^28^	ND	Ob 11 (100%) Lean 12 (100%)	41 ± 5 44 ± 9	Up	USA^31^	Fried Frailty Phenotype	Fr 7 (0%) N‐F 7 (71%)	Range 71–89 85.6 ± 3.8 76 ± 6.5	3.23
15	MiR‐196a‐5p	Singapore^29^	BMI ≥ 27.5 kg/m^2^	Ob 9 (0%) Lean 9 (0%)	28.4 ± 1.6^a^ 23.2 ± 0.2^a^	0.885	USA^31^	Fried Frailty Phenotype	Fr 7 (0%) N‐F 7 (71%)	Range 71–89 85.6 ± 3.8 76 ± 6.5	1.48
16	MiR‐296‐3p	Singapore^29^	BMI ≥ 27.5 kg/m^2^	Ob 9 (0%) Lean 9 (0%)	28.4 ± 1.6^a^ 23.2 ± 0.2^a^	1.049	USA^31^	Fried Frailty Phenotype	Fr 7 (0%) N‐F 7 (71%)	Range 71–89 85.6 ± 3.8 76 ± 6.5	0.20
17	MiR‐29b‐2‐5p	New Zealand^28^	ND	Ob 11 (100%) Lean 12 (100%)	41 ± 5 44 ± 9	ND (Up)	USA^31^	Fried Frailty Phenotype	Fr 7 (0%) N‐F 7 (71%)	Range 71–89 85.6 ± 3.8 76 ± 6.5	3.68
18	MiR‐301b‐3p	Singapore^29^	BMI ≥ 27.5 kg/m^2^	Ob 9 (0%) Lean 9 (0%)	28.4 ± 1.6^a^ 23.2 ± 0.2^a^	−0.973	USA^31^	Fried Frailty Phenotype	Fr 7 (0%) N‐F 7 (71%)	Range 71–89 85.6 ± 3.8 76 ± 6.5	−1.00
19	MiR‐378c	Singapore^29^	BMI ≥ 27.5 kg/m^2^	Ob 9 (0%) Lean 9 (0%)	28.4 ± 1.6^a^ 23.2 ± 0.2^a^	0.676	USA^31^	Fried Frailty Phenotype	Fr 7 (0%) N‐F 7 (71%)	Range 71–89 85.6 ± 3.8 76 ± 6.5	2.41
20	MiR‐4732‐5p	Singapore^29^	BMI ≥ 27.5 kg/m^2^	Ob 9 (0%) Lean 9 (0%)	28.4 ± 1.6^a^ 23.2 ± 0.2^a^	0.88	USA^31^	Fried Frailty Phenotype	Fr 7 (0%) N‐F 7 (71%)	Range 71–89 85.6 ± 3.8 76 ± 6.5	2.97
21	MiR‐487a‐3p	Singapore^29^	BMI ≥ 27.5 kg/m^2^	Ob 9 (0%) Lean 9 (0%)	28.4 ± 1.6^a^ 23.2 ± 0.2^a^	−1.3	USA^31^	Fried Frailty Phenotype	Fr 7 (0%) N‐F 7 (71%)	Range 71–89 85.6 ± 3.8 76 ± 6.5	−1.03
22	MiR‐766‐3p	New Zealand^28^	ND	Ob 11 (100%) Lean 12 (100%)	41 ± 5 44 ± 9	ND (Up)	USA^31^	Fried Frailty Phenotype	Fr 7 (0%) N‐F 7 (71%)	Range 71–89 85.6 ± 3.8 76 ± 6.5	1.14
	**Serum**										
23	MiR‐23a‐3p	Korea^25^	BMI ≥ 35 kg/m^2^	Ob 16 (56%) Lean 18 (72%)	Range 30–59 31.3 ± 8.76 38.6 ± 7.9	2.81	USA^34^	EWGSOP 2010	S 12 (100%) N‐S 51 (100%)	Range 60–85 ND ND	1.66 (NS)
	**Vastus lateralis**										
24	MiR‐424‐5p	ND^35^	ND	Ob 5 (0%) Lean 5 (0%)	ND	ND (Up)	UK^33^	EWGSOP 2010	S 5 (0%) N‐S 59 (0%)	Range 68–76	Up

AWGS, Asian Working Group for Sarcopenia; BMI, body mass index; EWGSOP, European Working Group on Sarcopenia in Older People; Fr, frail; IQR, inter‐quartile range; ND, not documented; N‐F, non‐frail; N‐S, non‐sarcopenic; NS, not statistically significant; Ob, obese; S, sarcopenic; SD, standard deviation; SEM, standard error of the mean.

Age is presented as mean ± SD unless specified.

^a^ ±SEM.

^b^ Median ± IQR.

Two miRNAs may also be commonly expressed in obesity and sarcopenia, but differences in nomenclature limited our understanding. Plasma miR‐328‐3p^29^ and miR‐328^28^ were down‐regulated and up‐regulated in obesity, respectively, and miR‐328 was down‐regulated in sarcopenia.^32^ Plasma miR‐215 was up‐regulated in both obesity^28^ and sarcopenia,^31^ and miR‐215‐5p was also up‐regulated in obesity.^29^ However, as we could not determine whether miR‐215 was ‐3p or ‐5p using the previous ID section on miRBase, we classified this miRNA as an unclear match. A list of the top externally validated circulating (plasma or serum) miRNAs in obesity or sarcopenia is available in *Table* S3.

### Assessment of risk of bias

The majority of studies scored ≤6 on the NOS (2,^25^ 3,^28^, ^35^ 4,^31^ 5,^29^ and 6,^33^, ^34^), two received a star for validation (4*^27^ and 6*^30^), and one study scored >6 (8*^32^). All studies lost a mark for failing to comment on the representativeness of cases,^25^, ^27^, ^28^, ^29^, ^30^, ^31^, ^32^, ^33^, ^34^, ^35^ five of six obesity studies lost a mark for failing to adequately define how controls were selected,^25^, ^27^, ^28^, ^29^, ^35^ and only four studies received marks for adequately describing how exposure was ascertained.^29^, ^32^, ^33^, ^34^ All studies, except three,^27^, ^29^, ^35^ received a mark for adequately describing the case definition.

### Validated target genes, metabolic pathways, and functions of microRNAs

Validated target genes of the miRNAs of interest, in relation to sarcopenia, obesity, and related conditions (e.g. insulin resistance, inflammation, and cachexia) where possible, were identified by conducting a literature search using PubMed (*Table* 2). The majority of validated targets identified in the literature search were members of the phosphoinositide 3‐kinase/protein kinase B (PI3K/AKT) and transforming growth factor‐β (TGF‐β) signalling pathways. The most common targets identified were IGF‐1 (miR‐424‐5p, miR‐483‐3p, and miR‐18b‐5p) and members of the SMAD family (miR‐483‐3p, miR‐92a‐3p, and miR‐424‐5p). MiRNAs also targeted phosphatase and tensin homologue (PTEN) (miR‐296‐3p and miR‐499) and peroxisome proliferator‐activated receptor‐γ coactivator 1‐α (PGC‐1α) both directly (miR‐23a‐3p) and indirectly (miR‐499 via Fnip). The forkhead box protein (FOXO) family was targeted by miR193b‐5p. AMP‐activated protein kinase (AMPK) was also targeted directly (miR‐1224‐5p) and indirectly (miR‐499 via Fnip). The majority of commonly expressed miRNAs were involved in protein homeostasis, mitochondrial dynamics, determination of muscle fibre type, insulin resistance, and adipogenesis.

**Table 2:** Functions and predicted targets of miRNAs that are differentially expressed in the same direction in obesity and sarcopenia

MiRNA (family)	Cluster	↑↓	Function in relation to obesity/adiposity/insulin resistance or sarcopenia/muscle/exercise	Sample	Target
**Plasma**					
MiR‐106a‐5p (miR‐17)	MiR‐106a, miR‐18b, miR‐20b, miR‐19b‐2, miR‐92a‐2, miR‐363	↑	Down‐regulated in polycystic ovary syndrome (PCOS)^36^ Elevated in aged muscles (mice) and dexamethasone‐treated myotubes; agomir results in down‐regulation of both myogenic regulatory factors (MyoD, MyoG, and MyHC) and phosphorylation of AKT and decreased myotube size^37^	Plasma exosomes^36^ C2C12 cells^37^ Mice^37^	PIK3R1^37^
MiR‐1224‐5p (miR‐1224)	N/A	↑	Up‐regulated in the liver of obese and high‐fat diet‐fed mice, contributes to hepatic lipid accumulation by targeting AMPKα1^38^	Mice^38^	AMPKα1^38^
MiR‐1246 (miR‐1246)	N/A	↑	Down‐regulated in patients with chronic obstructive pulmonary disease (COPD) and emphysema (*n* = 20)^39^ and amyotrophic lateral sclerosis (ALS) patients (*n* = 14)^40^ Up‐regulated in diabetic nephropathy patients (*n* = 23); positively correlated with BMI^41^	Serum^39^, ^41^ Plasma^40^	
MiR‐145‐5p (miR‐145)	MiR‐145, miR‐143	↑	Limited studies on obesity/sarcopenia Up‐regulated in normal‐weight women (*n* = 11) following a high‐energy/fat breakfast^42^	Plasma^42^	
MiR‐18b‐5p (miR‐17)	MiR‐106a, miR‐18b, miR‐20b, miR‐19b‐2, miR‐92a‐2, miR‐363	↑	Limited studies on obesity/sarcopenia Up‐regulated in PCOS^43^ and relapsing multiple sclerosis (MS), may be involved in inflammatory pathways^44^ SORBS2 identified as a target in diabetic nephropathy model cells^45^ Targets and inhibits IGF‐1, suppressing the activation of p‐AKT, p‐MEK, and p‐ERK1/2 *in vitro* ^46^	Serum^43^, ^44^ HGMCs/HRGECs^45^ HRECs^46^	SORBS2^45^ IGF‐1^46^
MiR‐193b‐5p (miR‐193)	MiR‐193b, miR‐365a	↑	Limited studies on obesity/sarcopenia Weak negative correlations with BMI, plasma glucose levels, and insulin response to OGTT in younger adults^47^ Targets and decreases expression of FoxO3 in cells, regulating cell cycle and cell proliferation^48^	Subcutaneous adipose tissue^47^ BRL‐3A^48^	FoxO3^48^
MiR‐196a‐5p (miR‐196)	N/A	↑	High level of expression in myoblasts, suppresses mitochondrial biogenesis and its master regulator, PGC1β, and ND4. Suppresses osteoclast formation induced by RANKL in Raw264.7 cells^49^	C2C12 cells^49^ Raw264.7 cells^49^	
MiR‐197‐3p (miR‐197)	N/A	↑	Increased after high‐intensity resistance exercise in young adults^50^ Up‐regulation inhibits GIP and GLP‐1 production through suppression of PCSK1/3^51^	Serum^50^ STC‐1 cells^51^	
MiR‐199a‐5p (miR‐199)	MiR‐214	↓	Overexpression of AKT down‐regulates miR‐199a‐5p with a subsequent increase in targets Sirt1 and HiF‐1α in cardiomyocytes^52^ Down‐regulated in mild and terminal‐stage ALS^53^ and patients with Parkinson's disease^54^ Up‐regulated in middle‐aged adults with T2DM; *in vitro* studies showed that miR‐199a regulates cellular glucose uptake by targeting and suppressing GLUT4^55^ Up‐regulated in rat pancreatic β‐cells exposed to high glucose, promotes apoptosis and ROS formation, suppresses SIRT1^56^ Inhibition results in decreased myogenic differentiation and increased MyoD1 and Pax7 in human myoblasts. High levels inhibit WNT signalling in HEK293T cells. Overexpression in zebrafish results in disorganization and detachment of myofibres^57^	Cardiomyocytes^52^ Serum^53^ Plasma^55^ Induced pluripotent stem cells^54^ Rat pancreatic β‐cells^56^ Myoblasts, HEK293T cells, zebrafish^57^	Sirt1^52^, ^56^, ^58^ HiF‐1α^52^ GLUT4^55^
MiR‐296‐3p (miR‐296)	MiR‐296, miR‐298	↑	Up‐regulated in PCOS; reduction in miR‐296‐3p promotes cell proliferation^59^	Human granulosa cells^59^ Human granulosa‐like tumour cells^59^	PTEN^59^
MiR‐29b‐2‐5p (miR‐29)	MiR‐29b‐2, miR‐29c	↑	Limited studies in the context of muscle/obesity Targets STAT3 in a fibroblast cell line^60^	L929 cells^60^	STAT3^60^
MiR‐301b‐3p (miR‐130)	MiR‐301b, miR‐130	↓	Decreased during myogenic differentiation; may be involved in muscle differentiation by regulating Rb1cc1^61^	Chicken myoblasts^61^	Rb1cc1^61^
MiR‐378c	N/A	↑	Studies not identified in the context of muscle/obesity		
MiR‐4732‐5p (miR‐4732)	MiR‐4732, miR‐144, miR‐451a, miR‐451b	↑	Studies not identified in the context of muscle/obesity		
MiR‐483‐3p (miR‐483)	N/A	↑	Up‐regulated in hyperglycaemic mice and cardiomyocytes. Overexpression down‐regulates IGF‐1, thus promoting apoptosis in hyperglycaemic cardiomyocytes^62^ Overexpression inhibits bovine myoblast cell proliferation through the *IGF1/PI3K/AKT* pathway; knockdown of miR‐483 enhances the expression of myogenic maker genes *MyoD1*, *MyoG*, and *MyHC* ^63^ Elevated in Duchenne's muscular dystrophy^64^	Mice, H9c2 cell line^62^ Bovine myoblasts^63^ Serum^64^	IGF‐1^62^, ^63^
MiR‐487a‐3p (miR‐154)	MiR‐1185‐1, miR‐1185‐2, miR‐381, miR‐487a, miR‐487b, miR‐539, miR‐889, miR‐544a, miR‐655, miR‐382, miR‐154, miR‐496, miR‐377, miR‐134, miR‐668, miR‐485, miR‐323b	↓	Studies not identified in the context of muscle/obesity		
MiR‐499a (miR‐499) *MyomiR*	MiR‐499a, miR‐499b Encoded in slow myosin heavy chain genes (*Myh7b*)—restricted to T1 fibres (expressed in T1 fibres only)	↓	Elevated in patients and carriers (mothers) with Duchenne's muscular dystrophy^65^ and COPD (*n* = 103) and significantly correlated with NF‐κB p50^66^ Affected by aerobic exercise—no changes after acute bout in young men^67^; decreased following acute bout with weight vest with/without nutritional supplementation^68^; increased in male marathon runners (*n* = 21) after competitive marathon competition^69^ Increased after essential amino acid (EAA) ingestion in young adults (*n* = 7)^70^ Associated with a slow muscle fibre phenotype in human muscle^71^ Double knockout miR‐499/miR‐208b mice lost slow Type I myofibres with a concomitant increase in fast Type IIx/d and IIb myosin isoforms; forced expression of miR‐499 converted fast myofibres to slow. Sox6 helps mediate the actions of miR‐499 on slow myofibre gene programming^72^ Targets Thrap1 to promote slow muscle fibre type^73^ Targets TGF‐βR1, a known regulator of skeletal myoblast development. Knockdown of TGF‐βR1 inhibits myogenic differentiation in C2C12 cells^74^ Targets PRDM16, which subsequently promotes myogenic, rather than brown adipogenic, differentiation in mouse skeletal muscle stem cells (SMSCs)^75^ Promotes mitochondrial function. Targets Fnip1, a negative regulator of mitochondrial function in myocytes, which leads to activation of PGC‐1α. Fnip1 inhibition stimulates oxygen consumption rates, a sign of mitochondrial function, in myocytes. Mice with muscular dystrophy bred with miR‐499 mice exhibit improved mitochondrial capacity, restored slow‐oxidative muscle fibre programming and greater muscle functionality assessed with treadmill distance^76^ Knockdown of p21, a target of miR‐499, decreases mitochondrial fission and cell death in cardiomyocytes exposed to doxorubicin, anti‐tumour drug^77^ PTENP1, a target gene of miR‐499, expression is enhanced in diabetic and obese mouse models resulting in impaired AKT/GSK activation and glycogen synthesis contributing to insulin resistance^78^ Down‐regulation was observed in diabetic mouse models. Down‐regulation *in vitro* was shown to impair the insulin signalling, AKT/GSK pathway and glycogen synthesis. PTEN was identified as a target^79^	Plasma^65^, ^66^, ^69^ Serum^67^ Vastus lateralis^68^, ^70^, ^71^ Mice^71^, ^72^, ^76^, ^77^, ^78^, ^79^ C2C12 cells^72^, ^73^, ^74^ SMSCs^75^ H9c2 cells^77^ Murine liver cells NCTC1469^78^, ^79^	Sox6^72^ Thrap1^72^, ^73^ p21^77^ TGF‐βR1^74^ PRDM16^75^ Fnip1^76^ PTEN^79^ PTENP1^78^
MiR‐550a‐3p (miR‐550)	MiR‐550a‐1, miR‐550b‐1	↑	Limited studies in muscle/obesity Down‐regulated in patients with sporadic ALS^80^ Associated with parameters of bone formation and microstructure parameters (mineral apposition ratio, bone surface, trabecular bone volume)^81^ Down‐regulated in postmenopausal women with fractures older than 6 months; excellent discrimination of patients with low traumatic fractures^82^	Peripheral blood^80^ Serum^81^, ^82^	
MiR‐576‐5p (miR‐576)	N/A	↑	Studies not identified in the context of muscle/obesity		
MiR‐589‐5p (miR‐589)	N/A	↑	Limited studies in muscle/obesity Decreased upon TGF‐β stimulation in control fibroblasts, with no effect seen in COPD fibroblasts^83^	Fibroblasts^83^	
MiR‐766‐3p (miR‐766)	N/A	↑	Decreased in older (60–73 years; *n* = 51) compared with younger (19–42 years; *n* = 55) or long‐lived (90–102 years; *n* = 51) adults.^84^ Overexpressed in older adult human dermal fibroblasts (HDFs)^85^ Decreased after 12 weeks of endurance training in young men (*n* = 32)^86^ Increased in sedentary T2DM adults (40–70 years; *n* = 24) who undertook either 4 month resistance or aerobic training^87^	PBMCs^84^ HDFs^85^ HeLa cells^85^ Plasma^86^, ^87^	SIRT6^85^
MiR‐92a‐3p (miR‐92a)	MiR‐17, miR‐18a, miR‐19a, miR‐20a, miR‐19b‐1, miR‐92a‐1	↑	Anti‐miR, MRG‐110, was tested in adult men and found to counteract the repression of known miR‐92a‐3p targets, ITGA5 and CD93. Elevated levels of DDIT4, an inhibitor of mTOR, were found in cells treated with MRG‐110^88^ In a systematic review, down‐regulated following bariatric surgery^89^ Decreased following 20 week aerobic exercise training (*n* = 20),^90^ 12 week endurance training in young men (*n* = 32),^86^ and a 6 week cycling training in young men (*n* = 24)^91^ No change following 5 month aerobic training in obese older adults (*n* = 33); however changes in miR‐92a positively correlated with changes in gait speed following intervention^92^ MiR‐92a targets SMAD7, inhibition of miR‐92a led to increased mitochondrial content and oxygen consumption of brown adipocytes; inhibition of miR‐92a led to promotion of SMAD7 and subsequent suppression of p‐SMAD3/SMAD3. Inhibition of miR‐92a promoted differentiation of brown adipocytes.^93^ Negatively correlated with BAT activity in young adults (*n* = 41); down‐regulated in the serum exosomes of mice with active BAT^94^ Gradually up‐regulated with age (22, 40, 59, and 70 years) in men and women^95^	Whole blood^88^ CD4^+^ T cells^88^ Plasma^86^, ^89^, ^92^ Serum^90^, ^94^, ^95^ C2C12 cells^93^ Vastus lateralis^91^ Mice^94^	ITGA5^88^ CD93^88^ SMAD7^93^
**Serum**					
MiR‐23a‐3p (miR‐23)	Mir‐23a, miR‐27a, miR‐24‐2	↑	Significantly down‐regulated in SAT and VAT of obese participants and significantly correlated with measures of adiposity (BMI, waist circumference, insulin measures). Involved in the regulation of PTEN, although the molecular mechanism is unclear^96^ In young men (*n* = 7), increased following resistance or endurance exercise and protein ingestion^97^ Increased following EAA ingestion alone^70^ Decreased after an acute bout of endurance exercise in young adults (*n* = 9)^98^ Up‐regulated in ALS. Targets PGC‐1α with subsequent effects on mitochondrial biogenesis and activity^99^ Protects muscles from atrophy by targeting atrogin‐1/MAFbx1 and MURF‐1. Overexpression counteracts muscle atrophy induced by dexamethasone in myotubes and glucocorticoids in mice^100^	VAT, SAT^96^ Vastus lateralis^70^, ^98^, ^99^ Mice^99^, ^100^ Adipocytes^96^ C2C12 cells^100^	Atrogin‐1/MAFbx1^100^ MURF‐1^100^ PGC‐1α^99^, ^101^
**Vastus lateralis**					
MiR‐424‐5p (miR‐322)	MiR‐424, miR‐503, miR‐542, miR‐450a‐2, miR‐450a‐1, miR‐450b	↑	Down‐regulated in young women with PCOS (*n* = 24).^43^ No difference between obese (*n* = 21) and NW (*n* = 19) women but correlated with waist circumference^102^ Increased in cachectic cancer patients^103^ Up‐regulated in muscle wasting conditions—ICU‐acquired weakness and COPD. Overexpression causes a reduction in muscle diameter of mice^33^ Saturated fat/high‐fat diet impairs insulin signalling (INSR and IRS‐1) and up‐regulated miR‐424‐5p in hepatocytes and mice. Overexpression causes a significant decrease in insulin‐induced glycogen synthesis in hepatocytes. INSR is a direct target^104^ Targets IGF‐1 in mice and human myocytes^105^	Serum^43^ Mice^33^, ^104^ Vastus lateralis^33^, ^103^ SAT^102^ Plasma^102^ Hepatocytes^104^ C2C12 cells^105^ Human myoblasts^105^	SMAD7^33^ INSR^104^ IGF‐1^105^

↑, up‐regulated in sarcopenia/obesity; ↓, down‐regulated in sarcopenia/obesity; HGMCs, human glomerular mesangial cells; HRECs, human retinal endothelial cells; HRGECs, human renal glomerular endothelial cells; PBMCs, peripheral blood mononuclear cells.

## Discussion

In this systematic review, we identified 24 miRNAs that are differentially expressed in both sarcopenia and obesity. These findings are particularly novel as miRNAs have not yet been explored in the context of sarcopenic obesity. The common dysregulation of the miRNAs identified in this review may therefore provide clues to understand the pathogenesis of sarcopenic obesity. To address this aim, a search was subsequently undertaken to understand the functions of these 24 miRNAs in relation to muscle/sarcopenia and adiposity/obesity. For some miRNAs, there were limited or no studies in the context of obesity or sarcopenia, and therefore, their relevance in relation to sarcopenic obesity is still unclear at present (miR‐29b‐2‐5p, miR‐378c, miR‐4732‐5p, miR‐487a‐3p, miR‐550a‐3p, miR‐576‐5p, and miR‐589‐5p). Other miRNAs have been shown to be differentially regulated in related diseases or metabolic responses, for example, in chronic obstructive pulmonary disease or amyotrophic lateral sclerosis (miR‐1246),^39^, ^40^ in response to a high‐fat meal (miR‐145‐5p)^42^ or exercise (miR‐766‐3p).^86^, ^87^ However, we found that the majority of these commonly expressed miRNAs were involved in protein homeostasis, mitochondrial dynamics, determination of muscle fibre type, insulin resistance, and adipogenesis—processes implicated in the development of sarcopenic obesity.^2^, ^9^ The targets identified were predominantly found in the PI3K/AKT and TGF‐β pathways.

### Protein homeostasis

IGF‐1 is one of the most important mediators of muscle growth and repair^106^; however, IGF‐1 declines with age.^107^ MiRNAs identified to be up‐regulated in both obesity and sarcopenia target IGF‐1, leading to its inhibition. The miRNAs miR‐18b‐5p, miR‐483‐3p, and miR‐424‐5p target IGF‐1 *in vitro*.^46^, ^62^, ^63^, ^105^ Functionally, *in vitro* studies have shown that miR‐483‐3p inhibits bovine myoblast cell proliferation through the IGF1/PI3K/AKT pathway^63^ and promotes apoptosis in hyperglycaemic cardiomyocytes.^62^ Up‐regulation of miR‐483‐3p causes a reduction in muscle diameter in mice and is also up‐regulated in muscle wasting conditions in humans.^33^ These studies therefore suggest that up‐regulation of these miRNAs could be detrimental to muscle metabolism.

We found that miRNAs implicated in both obesity and sarcopenia regulate several targets of the PI3K/AKT pathway, which is involved in protein homeostasis.^108^ Downstream of AKT, FoxO3 is targeted by miR‐193b‐5p.^48^ The FOXO family is implicated in many processes included cell cycle, apoptosis, autophagy, and muscle atrophy.^48^, ^108^ In muscle, FOXO proteins are important mediators of two major proteolytic cellular pathways—the autophagy–lysosome and ubiquitin–proteasome systems.^108^ These pathways are critical for quality control of sarcomeric proteins.^108^ Interestingly, miR‐23a‐3p targets the muscle atrophy genes atrogin‐1/MAFbx1 and MURF‐1, which are downstream targets of FOXO.^100^ Ectopic overexpression of miR‐23a‐3p counteracts muscle atrophy in dexamethasone‐treated myotubes and glucocorticoid‐treated mice.^100^ In addition to direct targeting, miRNAs have been reported to target regulators of the FOXO family. MiR‐199a‐5p, which is down‐regulated in sarcopenia and obesity, targets and suppresses Sirt1,^56^, ^58^ which is responsible for the deacetylation of FOXO; suppression of Sirt1 results in cellular senescence *in vitro*.^58^ Up‐regulation of miR‐199a‐5p promotes apoptosis and ROS formation *in vitro*.^56^ Dysregulation in the PI3K/AKT/FOXO pathway may have implications on muscle atrophy and muscle quality control with implications for sarcopenia.

In mature adult muscle, TGF‐β is a potent regulator of muscle atrophy, which impairs skeletal muscle regeneration through inhibition of satellite cell proliferation, myofibre fusion, and expression of some muscle‐specific genes.^109^ Multiple miRNAs commonly expressed in sarcopenia and obesity target this pathway. MiR‐499 targets TGF‐βR1, a receptor for TGF‐β.^74^ Knockdown of this receptor inhibits myogenic differentiation in C2C12 cells.^74^ Downstream of TGF‐βR1, Smad4 is down‐regulated by miR‐483‐3p to induce apoptosis *in vitro*.^110^ Two miRNAs up‐regulated in obesity and sarcopenia, miR‐424‐5p^33^ and miR‐92a‐3p,^93^ target Smad7, a strong inhibitor of the TGF‐β pathway, which in turn inhibits SMAD2/3. Therefore, in sarcopenia and obesity, the TGF‐β pathway may be inhibited by miR‐483‐3p but promoted by miR‐424‐5p, miR‐92a‐3p, and the down‐regulation of miR‐499. It is unclear what effect this may have in relation to the pathogenesis of sarcopenic obesity, but known targets of TGF‐β pathway include the muscle atrophy genes, atrogin‐1 and MuRF‐1.

### Mitochondrial dynamics

MicroRNAs dysregulated in both obesity and sarcopenia regulate mitochondrial biogenesis. MiR‐196a‐5p is highly expressed in myoblasts and suppresses PGC1β, a regulator of mitochondrial biogenesis.^49^ MiR‐499a‐5p, which is down‐regulated in sarcopenia/obesity, targets Fnip1, which in turn inhibits AMPK with subsequent reduced activation of PGC‐1α.^76^ Functionally, inhibition of Fnip1 by miR‐499a‐5p results in improved mitochondrial function in myocytes and improved mitochondrial capacity in mice with muscular dystrophy.^76^ In adults with amyotrophic lateral sclerosis, of which mitochondrial dysfunction is considered an important factor in its pathogenesis, miR‐23a‐3p is elevated^99^ similar to adults with obesity and sarcopenia.^25^, ^34^ MiR‐23a‐3p targets PGC‐1α with inhibition of its downstream signalling of mitochondrial biogenesis.^99^ Likewise, miR‐92a‐3p^93^ and miR‐424‐5p^33^ target SMAD7, an antagonist of SMAD2/3. Functionally, miR‐92a‐3p inhibits mitochondrial content and oxygen consumption of brown adipocytes.^93^ MiR‐499a‐5p also inhibits mitochondrial fission and apoptosis in cardiomyocytes exposed to an anti‐tumour drug by targeting p21, thus preventing cardiotoxicity.^77^ Taken together, the dysregulation observed in these miRNAs in obesity and sarcopenia may lead to impaired mitochondrial function.

### Fibre‐type switching

Ageing is associated with a switch from a fast muscle fibre phenotype to one of a slow muscle fibre type,^111^ whereas obesity is associated with a greater proportion of fast, Type II, muscle fibres.^112^ Overexpression of miR‐499 *in vitro* is associated with conversion of fast myofibres to slow through targeting of Sox6 and Thrap1.^72^, ^73^ In mice, knockout of miR‐499 and miR‐208b results in a loss of slow Type I myofibres and an increase in fast Type IIx/d and IIb myofibres.^72^ In humans, miR‐499 is associated with a slow muscle fibre phenotype^71^ and is elevated in patients with Duchenne's muscular dystrophy^65^ and chronic obstructive pulmonary disease.^66^ It is interesting that miR‐499 is underexpressed in both sarcopenia and obesity in light of the different muscle fibre properties of obesity and sarcopenia. However, it must be noted that this miRNA was reported in plasma rather than skeletal muscle where levels may be different.

### Insulin resistance

Obesity is associated with insulin resistance, which can also impair muscle regeneration.^2^, ^12^ Several miRNAs identified as commonly up‐regulated in sarcopenia and obesity affect glucose metabolism and are associated with insulin resistance.^47^, ^51^, ^104^ MiR‐197‐3p can regulate glucose metabolism by suppressing PCSK1/3 to inhibit GIP and GLP‐1 production, incretin hormones implicated in the pathogenesis of diabetes.^51^ Overexpression of miR‐424‐5p is associated with decreased insulin‐induced glycogen synthesis in hepatocytes.^104^ In young adults, miR‐193b‐5p is also negatively correlated with BMI, plasma glucose levels, and insulin response.^47^ In contrast, down‐regulation of two miRNAs in obesity and sarcopenia may be beneficial for glucose metabolism—miR‐199a‐5p and miR‐499.^55^, ^78^ MiR‐199a‐5p is up‐regulated in diabetes, and *in vitro* studies have shown that miR‐199a‐5p targets and represses GLUT4, a glucose transporter isoform that increases glucose transport in response to insulin.^55^ MiR‐499 targets both PTEN and PTENP1; PTENP1 can act as a ‘sink’ for miR‐499 to allow glucose metabolism.^78^ It is unclear what effect these miRNAs may have in relation to the pathogenesis sarcopenic obesity.

### Adiposity and adipogenesis

Gains in body fat and intramyocellular lipid deposition are characteristic of ageing, obesity, and sarcopenia.^2^, ^10^, ^111^ MiRNAs differentially expressed in both obesity and sarcopenia were associated with parameters of adiposity. Body mass index is correlated with miR‐1246^41^ and miR‐193b‐5p^47^ in adults. MiR‐92a is negatively correlated with brown adipose tissue activity in young adults,^94^ and inhibition of miR‐92a up‐regulates brown adipocyte differentiation *in vitro*.^93^ MiR‐499a‐5p promotes myogenic rather than adipogenic differentiation in skeletal muscle stem cells.^75^ MiR‐1224‐5p contributes to hepatic lipid accumulation in mice by targeting AMPKα1.^38^

### Limitations

The limitations of this study must be considered. Firstly, the heterogeneity and low quality of the studies identified in this review must be acknowledged. In some cases, matches were found between younger or female obese studies and older or predominantly male sarcopenia studies. As such, it is unclear how the interaction of age or sex has impacted our findings. It is known that age affects miRNA profiles, so perhaps older obese adults have differing miRNA profiles than younger obese adults, likewise men and women may exhibit differing profiles within the same condition. Secondly, we only included studies that had significantly different miRNAs, and therefore, we may have missed studies with non‐significant miRNAs, which may dispute our findings. However, this approach is commonly accepted.^113^, ^114^ Thirdly, because of the large number of overlapping miRNAs identified, we chose to discuss miRNAs, which were commonly dysregulated in the same direction in both conditions. The overlapping miRNAs identified were not externally validated, and therefore, our results should be viewed with caution and in need of further validation. MiRNAs that were reported as being expressed in conflicting directions in obesity may be due to differences between study methodology. Therefore, these miRNAs also warrant consideration in future research. Because of the large number of overlapping miRNAs, we chose to search for functions and targets in the context of obesity and sarcopenia and therefore may have omitted findings from other conditions, which may be relevant to sarcopenic obesity. However, a strength of our approach is that we focused on sarcopenia, obesity, and related conditions or diseases to focus our narrative review. It is possible that frailty and sarcopenia have differing miRNA profiles; however, because of limited studies and a similar clinical manifestation, it was deemed that information available on frailty may be useful in this context. In addition, the majority of miRNAs identified in sarcopenia/frailty were found in exosomes. There is evidence to suggest that some miRNAs appear to be preferentially recruited to exosomes whereas others are retained within the original cell.^115^ However, because of a limited number of studies conducted in sarcopenia, we therefore opted to use a more open definition and a less specific outcome measure to avoid missing potentially relevant findings.

## Conclusions

The pathogenesis underlying sarcopenic obesity is not fully understood. This is the first study to examine the potential role of miRNAs in the context of sarcopenic obesity and thus offers a novel perspective on this topic. We have provided an overview of the field and identified a panel of miRNAs, which may be implicated in sarcopenic obesity. Given the synergistic effect of sarcopenia and obesity on the risk of adverse health outcomes (falls, hospitalization, worsening disability, and all‐cause mortality), understanding the pathogenesis of sarcopenic obesity has the potential to lead to effective screening, monitoring, or treatment strategies. However, this systematic review was exploratory, and further work is now required to validate the findings presented here in older adults with sarcopenic obesity.

## Conflict of interest

None declared.

## Funding

K.G.‐W. is funded by Science Foundation Ireland (SFI) FFFP (19/FFP/6709), Irish Research Council (IRC) (IRCLA/2017/101), Health Research Board (HRB) (COV19‐2020‐060), and Dunhill Medical Trust (R545/0217). A.D. is funded by FIDELIO, an MSCA Innovative Training Network, receiving funding from the European Union's Horizon 2020 Framework Programme under Grant Agreement No. 860898. This work was supported by a studentship from the Medical Research Council (MRC) and Versus Arthritis as part of the Medical Research Council Versus Arthritis Centre for Integrated Research into Musculoskeletal Ageing (CIMA) (MR/R502182/1). The MRC Versus Arthritis Centre for Integrated Research into Musculoskeletal Ageing is a collaboration between the University of Liverpool, the University of Sheffield, and Newcastle University.

## Supporting information


**Table S1.** Eligible definitions/criteria for conditions studied.

**Table S2.** Interpretation of Newcastle‐Ottawa Quality Assessment Scale for Case Control Studies in the context of this study.

**Table S3.** Top externally validated circulating (plasma or serum) miRNAs in obesity and sarcopenia.
