# Supplementary material for: MicroRNAs in obesity, sarcopenia, and commonalities for sarcopenic obesity: a systematic review
Source: J Cachexia Sarcopenia Muscle. 2022 Jan 4;13(1):68–85. doi: 10.1002/jcsm.12878 (PMC8818592; doi:10.1002/jcsm.12878)
Supplement: Supplementary file 1 — Table S1. Eligible definitions/criteria for conditions studied. [file JCSM-13-68-s003.docx]

# Title Page

**Authors**

Lisa Dowling, BSc (Hons), MSc, MRes, The University of Sheffield

Ankita Duseja, BTech, MSc, The University of Sheffield

Tatiane Vilaca, MD, PhD, The University of Sheffield

Jennifer S Walsh, MBChB, PhD, FRCP, FHEA, The University of Sheffield

Katarzyna Goljanek-Whysall, BSc (Hons), MSc, PhD, The University of Liverpool and National University of Ireland, Galway, College of Medicine, Nursing and Health Sciences, School of Medicine, Department of Physiology.

**Title** MicroRNAs in obesity, sarcopenia and commonalities for sarcopenic obesity – a systematic review

**Journal name** Journal of Cachexia, Sarcopenia and Muscle

**Corresponding Author**

Lisa Dowling

Lmdowling1@sheffield.ac.uk

# Supporting Information Table S1: Eligible definitions/criteria for conditions studied

| **Condition** | **Definition** |
| --- | --- |
| Sarcopenia | Due to heterogeneous definitions, studies which define sarcopenia as low lean muscle mass, low muscle strength, or both will be included. |
| Frailty | - The use of gait speed (taking more than 5 seconds to walk 4 m using usual walking aids if appropriate) or Gait speed <0.8m/s  - Timed up and go test  - The PRISMA 7 questionnaire (with a cut-off score of >3)  - Clinical frailty scale  - Edmonton frail scale or Reported Edmonton frail scale  - Electronic Frailty Index (eFI)  - Fried criteria for frailty |
| Obesity | - High BMI  - High fat mass or % body fat mass  - High waist circumference  - High visceral fat area |
| Metabolic Syndrome | International Diabetes Federation Consensus Worldwide Definition of the Metabolic Syndrome (2017) |
