# Supplementary material for: MicroRNAs in obesity, sarcopenia, and commonalities for sarcopenic obesity: a systematic review
Source: J Cachexia Sarcopenia Muscle. 2022 Jan 4;13(1):68–85. doi: 10.1002/jcsm.12878 (PMC8818592; doi:10.1002/jcsm.12878)
Supplement: Supplementary file 2 — Table S2. Interpretation of Newcastle‐Ottawa Quality Assessment Scale for Case Control Studies in the context of this study. [file JCSM-13-68-s001.docx]

# Title Page

**Authors**

Lisa Dowling, BSc (Hons), MSc, MRes, The University of Sheffield

Ankita Duseja, BTech, MSc, The University of Sheffield

Tatiane Vilaca, MD, PhD, The University of Sheffield

Jennifer S Walsh, MBChB, PhD, FRCP, FHEA, The University of Sheffield

Katarzyna Goljanek-Whysall, BSc (Hons), MSc, PhD, The University of Liverpool and National University of Ireland, Galway, College of Medicine, Nursing and Health Sciences, School of Medicine, Department of Physiology.

**Title** MicroRNAs in obesity, sarcopenia and commonalities for sarcopenic obesity – a systematic review

**Journal name** Journal of Cachexia, Sarcopenia and Muscle

**Corresponding Author**

Lisa Dowling

Lmdowling1@sheffield.ac.uk

# Supporting Information Table S2: Interpretation of Newcastle-Ottawa Quality Assessment Scale for Case Control Studies in the context of this study

| **Selection** | **Interpretation** |
| --- | --- |
| 1) Is the case definition adequate? a) yes, with independent validation * b) yes, e.g. record linkage or based on self reports  c) no description | a) Refers to a consensus/validated/endorsed definition or uses the IDF definition for MetS, or clearly uses the WHO or Asia-Pacific cut-offs (Asian populations only) for BMI. |
| 2) Representativeness of the cases a) consecutive or obviously representative series of cases *  b) potential for selection biases or not stated | a) States that the participants have been recruited consecutively or are representative. |
| 3) Selection of Controls a) community controls *  b) hospital controls c) no description | a) States that the participants have been recruited from the community or are free living or it can be reasonable assumed that the participants are from the community  b) Participants who are inpatients or attending clinics  c) No description of how participants were recruited |
| 4) Definition of Controls a) no history of disease (endpoint) *  b) no description of source | a) Study either defines that participants did not have the disease (e.g. non-obese, lean), or indicates with the definition that the participants did not have the disease  b) No description of the definition for controls e.g. ‘healthy controls’ |
| **Comparability** |  |
| 1) Comparability of cases and controls on the basis of the design or analysis a) study controls for **age*** b) study controls for **gender** * | Study either states that the participants were matched based on age/gender or the study is e.g. female only. |
| **Exposure** |  |
| 1) Ascertainment of exposure a) secure record (eg surgical records) * b) structured interview where blind to case/control status *  c) interview not blinded to case/control status d) written self report or medical record only e) no description | a) Independently measured in the study  b-e) as described |
| 2) Same method of ascertainment for cases and controls  a) yes *  b) no | a) Same method of ascertainment can be assumed for both groups even if not explicitly said.  b) No description for ascertainment of exposure |
| 3) Non-Response rate a) same rate for both groups * b) non respondents described  c) rate different and no designation | Due to the study design, all studies were given a mark for this question. |
| **Additional - Validation** |  |
| 1) Validated  a) two methods (e.g. qPCR and RNA-seq) *  b) two separate study groups *  c) not validated | If a star is given, this is not included in the Newcastle-Ottawa Scale score rather as a “ * ” at the end of the score (e.g. 6*) |

**Abbreviations:** WHO = World Health Organisation, IDF = International Diabetes Federation, BMI = Body Mass Index
