# Supplementary material for: MicroRNAs in obesity, sarcopenia, and commonalities for sarcopenic obesity: a systematic review
Source: J Cachexia Sarcopenia Muscle. 2022 Jan 4;13(1):68–85. doi: 10.1002/jcsm.12878 (PMC8818592; doi:10.1002/jcsm.12878)
Supplement: Supplementary file 3 — Table S3. Top externally validated circulating (plasma or serum) miRNAs in obesity and sarcopenia. [file JCSM-13-68-s002.docx]

# Title Page

**Authors**

Lisa Dowling, BSc (Hons), MSc, MRes, The University of Sheffield

Ankita Duseja, BTech, MSc, The University of Sheffield

Tatiane Vilaca, MD, PhD, The University of Sheffield

Jennifer S Walsh, MBChB, PhD, FRCP, FHEA, The University of Sheffield

Katarzyna Goljanek-Whysall, BSc (Hons), MSc, PhD, The University of Liverpool and National University of Ireland, Galway, College of Medicine, Nursing and Health Sciences, School of Medicine, Department of Physiology

**Title** MicroRNAs in obesity, sarcopenia and commonalities for sarcopenic obesity – a systematic review

**Journal name** Journal of Cachexia, Sarcopenia and Muscle

**Corresponding Author**

Lisa Dowling

Lmdowling1@sheffield.ac.uk

# Supporting Information Table S3: Top externally validated circulating (plasma or serum) miRNAs in obesity and sarcopenia

|  | **miRNA** | **Count** | **Up** | **Down** |
| --- | --- | --- | --- | --- |
| **Obesity** | |  |  |  |
| 1 | miR-21-5p | 9 | [1-3] | [4-9] |
| 2 | miR-320a-3p | 6 | [10, 11] | [3, 12-14] |
| 3 | miR-122-5p | 6 | [1, 7, 15, 16] | [2, 8] |
| 4 | miR-221-3p | 6 | [2, 17] | [4, 6, 8, 13] |
| 5 | miR-126-3p | 5 | [2, 3, 8] | [6, 18] |
| 6 | miR-146a-5p | 5 | [2, 19] | [5, 6, 11] |
| 7 | miR-155-5p | 5 | [2, 20, 21] | [22, 23] |
| 8 | miR-192-5p | 5 | [1, 6, 7] | [2, 12] |
| 9 | miR-27a-3p | 5 | [1, 2, 14] | [6, 13] |
| 10 | miR-92a-3p | 5 | [1, 6, 10, 24] | [12] |
| **Sarcopenia** | |  |  |  |
| 1 | miR-23a-3p | 2 | [25, 26] |  |

The top 10 validated miRNAs are shown for obesity/metabolic syndrome. In sarcopenia, only one miRNA was reported by more than one study. Count refers to the number of separate studies reporting differential expression of the miRNA.

**References for Supporting Information Table S3:**

1. Bae YU, Kim Y, Lee H, Kim H, Jeon JS, Noh H, et al. Bariatric surgery alters microRNA content of circulating exosomes in patients with obesity. Obesity. 2019;27(2):264-271.

2. Jones A, Danielson KM, Benton MC, Ziegler O, Shah R, Stubbs RS, et al. miRNA signatures of insulin resistance in obesity. Obesity. 2017;25(10):1734-1744.

3. Yang P, Dong X,Zhang Y. MicroRNA profiles in plasma samples from young metabolically healthy obese patients and miRNA‐21 are associated with diastolic dysfunction via TGF‐β1/Smad pathway. J Clin Lab Anal. 2020;34(6):e23246. doi:10.1002/jcla.23246.

4. Assmann TS, Cuevas-Sierra A, Riezu-Boj JI, Milagro FI,Martínez JA. Comprehensive Analysis Reveals Novel Interactions between Circulating MicroRNAs and Gut Microbiota Composition in Human Obesity. Int J Mol Sci. 2020;21(24):9509. doi:10.3390/ijms21249509.

5. Benbaibeche H, Hichami A, Oudjit B, Haffaf EM, Kacimi G, Koceïr EA, et al. Circulating mir-21 and mir-146a are associated with increased cytokines and CD36 in Algerian obese male participants. Arch Physiol Biochem. 2020:1-6.

6. Choi H, Koh HW, Zhou L, Cheng H, Loh TP, Parvaresh Rizi E, et al. Plasma protein and microRNA biomarkers of insulin resistance: A network-based integrative-omics analysis. Front Physiol. 2019;10:379.

7. Sangiao-Alvarellos S, Theofilatos K, Barwari T, Gutmann C, Takov K, Singh B, et al. Metabolic recovery after weight loss surgery is reflected in serum microRNAs. BMJ Open Diabetes Research and Care. 2020;8(2):e001441. doi:10.1136/bmjdrc-2020-001441.

8. Ortega FJ, Mercader JM, Catalan V, Moreno-Navarrete JM, Pueyo N, Sabater M, et al. Targeting the circulating microRNA signature of obesity. Clin Chem. 2013;59(5):781-792.

9. Ghorbani S, Mahdavi R, Alipoor B, Panahi G, Nasli Esfahani E, Razi F, et al. Decreased serum microRNA-21 level is associated with obesity in healthy and type 2 diabetic subjects. Arch Physiol Biochem. 2018;124(4):300-305. doi:10.1080/13813455.2017.1396349.

10. Karolina DS, Tavintharan S, Armugam A, Sepramaniam S, Pek SLT, Wong MT, et al. Circulating miRNA profiles in patients with metabolic syndrome J Clin Endocrinol Metabl. 2012;97(12):E2271-E2276.

11. Santamaria-Martos F, Benitez ID, Latorre J, Lluch A, Moreno-Navarrete JM, Sabater M, et al. Comparative and functional analysis of plasma membrane-derived extracellular vesicles from obese vs. nonobese women. Clin Nutr. 2020;39(4):1067-1076.

12. Choi WH, Ahn J, Um MY, Jung CH, Jung SE,Ha TY. Circulating microRNA expression profiling in young obese Korean women. Nutr Res Pract. 2020;14(4):412-422.

13. Goguet-Rubio P, Klug RL, Sharma DL, Srikanthan K, Puri N, Lakhani VH, et al. Existence of a strong correlation of biomarkers and miRNA in females with metabolic syndrome and obesity in a population of West Virginia. Int J Med Sci. 2017;14(6):543.

14. Munetsuna E, Yamada H, Ando Y, Yamazaki M, Tsuboi Y, Kondo M, et al. Association of subcutaneous and visceral fat with circulating microRNAs in a middle-aged Japanese population. Ann Clin Biochem. 2018;55(4):437-445.

15. Kim H, Bae Y-U, Lee H, Kim H, Jeon JS, Noh H, et al. Effect of diabetes on exosomal miRNA profile in patients with obesity. BMJ Open Diabetes Research and Care. 2020;8(1):e001403.

16. Wang R, Hong J, Cao Y, Shi J, Gu W, Ning G, et al. Elevated circulating microRNA-122 is associated with obesity and insulin resistance in young adults. Eur J Endocrinol. 2015;172(3):291-300. doi:10.1530/EJE-14-0867.

17. Bao F, Slusher AL, Whitehurst M,Huang C-J. Circulating microRNAs are upregulated following acute aerobic exercise in obese individuals. Physiol Behav. 2018;197:15-21.

18. Manning P, Munasinghe PE, Bellae Papannarao J, Gray AR, Sutherland W,Katare R. Acute weight loss restores dysregulated circulating micrornas in individuals who are obese. J Clin Endocrinol Metab. 2019;104(4):1239-1248.

19. Russo A, Bartolini D, Mensà E, Torquato P, Albertini MC, Olivieri F, et al. Physical activity modulates the overexpression of the inflammatory miR‐146a‐5p in obese patients. IUBMB life. 2018;70(10):1012-1022.

20. Tryggestad JB, Teague AM, Sparling DP, Jiang S,Chernausek SD. Macrophage‐Derived microRNA‐155 Increases in Obesity and Influences Adipocyte Metabolism by Targeting Peroxisome Proliferator‐Activated Receptor Gamma. Obesity. 2019;27(11):1856-1864.

21. Zhu J, Wang C, Zhang X, Qiu T, Ma Y, Li X, et al. Correlation analysis of microribonucleic acid‐155 and microribonucleic acid‐29 with type 2 diabetes mellitus, and the prediction and verification of target genes. J Diabetes Investig. 2021;12(2):165-175.

22. Vonhögen IG, Mohseni Z, Winkens B, Xiao K, Thum T, Calore M, et al. Circulating miR-216a as a biomarker of metabolic alterations and obesity in women. Non-coding RNA Res. 2020;5(3):144-152.

23. Mahdavi R, Ghorbani S, Alipoor B, Panahi G, Khodabandehloo H, Esfahani EN, et al. Decreased serum level of miR-155 is associated with obesity and its related metabolic traits. Clin Lab. 2018;64(1):77-84. doi:10.7754/Clin.Lab.2017.170618.

24. Cereijo R, Taxerås SD, Piquer-Garcia I, Pellitero S, Martínez E, Tarascó J, et al. Elevated levels of circulating miR-92a are associated with impaired glucose homeostasis in patients with obesity and correlate with metabolic status after bariatric surgery. Obes Surg. 2020;30(1):174-179.

25. Ipson BR, Fletcher MB, Espinoza SE,Fisher AL. Identifying exosome-derived MicroRNAs as candidate biomarkers of frailty. J Frailty Aging. 2018;7(2):100-103. doi:10.14283/jfa.2017.45.

26. Chen Z, Bemben MG,Bemben DA. Bone and muscle specific circulating microRNAs in postmenopausal women based on osteoporosis and sarcopenia status. Bone. 2019;120:271-278.
